# Evaluating the utility of early laboratory monitoring of antiretroviral-induced haematological and hepatic toxicity in HIV-infected persons in Cameroon

**DOI:** 10.1186/1471-2334-14-519

**Published:** 2014-09-25

**Authors:** Cavin Epie Bekolo, Cecile Sonkoue, Hortense Djidjou, Patrick Sylvestre Bekoule, Basile Kollo

**Affiliations:** Centre Médical d’Arrondissement de Baré, P.O. Box 628, Nkongsamba, Cameroon; Regional Hospital of Nkongsamba, PO Box 03, Nkongsamba, Cameroon; Department of Public Health, University of Douala, PO Box 2701, Douala, Cameroon

**Keywords:** HIV, Antiretroviral therapy, Laboratory monitoring, Cameroon

## Abstract

**Background:**

The antiretroviral therapy (ART) program of Cameroon recommends routine laboratory monitoring of haematological toxicity if a regimen contains zidovudine (AZT) and of hepatotoxicity for NVP-containing regimens on the 15^th^ day after ART initiation. This study aimed to assess the relevance of this repeated laboratory measurements considered to be precocious, inaccessible and unavailable in a resource limited setting.

**Methods:**

A retrospective cohort of HIV-infected patients of age 15 years and above enrolled for first line ART at The Regional Hospital of Nkongsamba in Cameroon. We monitored liver transaminases and blood cell indices after two weeks of ART initiation for any significant change from baseline. Factors associated with abnormal changes were examined using a multivariable logistic regression model with random effects.

**Results:**

Enrolled were 154 patients of whom 105 (68.2%) were females. The mean ALAT (alanine aminotransferase) level at baseline was 17.87 ± 20.48 U/L increasing to 19.25 ± 12.01 U/L at two weeks of follow-up (p = 0.53) while the mean ASAT (aspartate aminotransferase) level increased from 17.32 ± 11.87 U/L at baseline to 21.02 ± 14.12 U/L at two weeks of follow-up (p = 0.02). We observed a drop in the mean haemoglobin concentration from 10.86 ± 2.63 g/dL at baseline to 10.36 ± 1.92 g/dL at the second week of follow-up (p = 0.02). The prevalence of elevated liver enzymes and anaemia after two weeks of treatment were 7.5% and 39.2% respectively. Stavudine containing regimens were most likely to induce hepatotoxicity [adjusted Odd Ratio (aOR) = 36.52, 95% CI: 1.44-924.38, p=0.029]. Baseline anaemia (aOR=60.08, 95% CI: 13.36-270.20, p < 0.0001) and body weight ≥ 60kg (aOR=0.28, 95% CI: 0.09-0.83, p = 0.02) were associated with anaemia at follow-up.

**Conclusion:**

There was no significant rise in the mean level of transaminases and thus scheduling their routine monitoring at the end of the second week could be skipped. Conversely, the drop in mean haemoglobin level had little clinical importance but the high prevalence of anaemia after a fortnight on treatment suggests a targeted instead of a routine monitoring; focusing on the high risk population with baseline anaemia and low body weight.

**Electronic supplementary material:**

The online version of this article (doi:10.1186/1471-2334-14-519) contains supplementary material, which is available to authorized users.

## Background

In 2012, 9.7 million people living with human immunodeficiency virus (HIV) in low- and middle-income countries received antiretroviral therapy (ART) [1]. Antiretroviral therapy does not only prevent acquired immune deficiency syndrome (AIDS)-related illness and death, it also has the potential to significantly reduce the risk of HIV transmission and the spread of tuberculosis. From 1996 to 2012, antiretroviral therapy averted 6.6 million AIDS-related deaths worldwide, including 5.5 million deaths in low- and middle-income countries [1]. However, ART is without adverse effects that may have deleterious consequences on the quality of care and treatment outcomes in the short or long term. Zidovudine (AZT) is associated with a risk of haematological toxicity, and measuring haemoglobin is recommended before initiating ART, mainly among adults and children with low body weight, low CD4 counts and advanced HIV disease [2]. Nevirapine (NVP) increases the risk of hepatotoxicity and monitoring hepatic enzymes is recommended if feasible, especially for women with HIV who have CD4 cell counts >250 cells/mm^3^ and individuals with HIV who are coinfected with hepatitis-B virus (HBV) or hepatitis-C virus (HCV) [2]. In order to determine and minimize the incidence of drug toxicity associated with the use of antiretroviral (ARV) medicines, The World Health Organisation (WHO) recommends a symptom-directed approach to laboratory monitoring of the safety and toxicity of ART regimens [2]. The periodicity of laboratory monitoring and the key types of toxicities are determined by various national ART programs. In Cameroon where the HIV prevalence is 4.3% [3] and the ART coverage is about 49.6% [4], the national guidelines in the light of WHO 2010 guidelines recommend amongst others: the maiden laboratory monitoring of haematological toxicity if a regimen contains AZT and hepatotoxicity for NVP-containing regimens on the 15^th^ day of ART initiation [5].

The laboratory measurement of liver enzymes has very low predictive value for NVP-containing regimens [2]; the incidence of ART-induced liver injury is low [6] and might even be a very rare occurrence after a fortnight of ART initiation; the cost of laboratory monitoring at the end of the second week of starting ART regimens is not subsidised and thus unlikely to be affordable to most patients in Cameroon. The financial burden associated with this early laboratory testing is likely to be a contributory factor for the high rates of early attrition in HIV care in Cameroon [7]. The increased work load linked to this monitoring further stretches the already constraint resources of the HIV program. Routine monitoring with multitest haematological and chemistry panels is unlikely to be cost-effective [8]. As the validity, feasibility and even the benefits of these precocious laboratory tests have therefore become uncertain; we sought to determine their current relevance in a resource constraint setting. We assessed if there were any significant pathological changes to the key haematological and biochemical markers from baseline to the 15^th^ day of follow-up as well as the determinants of such changes. We hoped that the findings of this study would contribute in updating the country’s current guidelines in line with the current WHO 2013 consolidated guidelines by selecting which laboratory tests to perform on the basis of their utility at a given point in time.

## Methods

### Study site

The study was conducted at the HIV clinic of the Regional Hospital of Nkongsamba, Moungo Division of the Littoral Region of Cameroon. It is a second level reference public health facility with a catchment area of over 321,295 inhabitants [9]. The HIV clinic was established in 2005 and offers HIV counselling and testing, ART and limited community outreach services to patients on ART. Routine monitoring of ART for hepatic and haematological toxicity at the 15^th^ day of ART is common practice.

### Ethical aspects

We used a database created in June 2012 as described elsewhere in a study that was approved by the London School of Hygiene and Tropical Medicine (LSHTM) Ethics Committee [7]. Onsite permission to conduct the study was provided by The Littoral Regional Office for Public Health in Cameroon and The Director of The Nkongsamba Regional Hospital. A written informed consent from participants or legal representatives was duly obtained where applicable.

### Study design, participants and data collection

We carried out between February and April 2014, a retrospective cohort of HIV-infected patients of age 15 years and above initiating first line ART and returning two weeks later for their first follow-up visit between 2005 and 2012. From the available database we abstracted the following information: socio-demographic characteristics which were date of birth, gender, place of residence, occupation, use of insecticide treated bed nets (ITN), alcohol and tobacco consumption, education and marital status. Clinical features such as baseline weight and height, pregnancy status, disease and clinical stage at presentation were also collected. Laboratory parameters assessed at baseline and at the end of the two week follow-up period included: haemoglobin level, mean red cell volume (MCV), total leucocyte count, total platelet count, liver transaminases which were alanine aminotransferase (ALAT) and aspartate aminotransferase (ASAT). Baseline CD4 cell counts and fasting blood sugar levels were also obtained. Treatment related variables such as the antiretroviral (ARV) regimen, drug side effects, fluconazole prophylaxis, cotrimoxazole prophylaxis, tuberculosis treatment, the use of haematinics and traditional medication were noted. These data were entered into a form designed using EpiInfo™ 7 software.

### Laboratory analysis

Liver enzymes were assayed using the semi-automated biochemistry analyser RAYTO RT-9900 (Hamburg, Germany) using commercial reagents (Hospitex Diagnostics, Firenze, Italy). Normal reference intervals for ALAT and ASAT according to the laboratory for Cameroonian subjects were (5–45 U/L) and (5–42 U/L), respectively. Full blood counts were obtained using the URIT 3300 Automated Haematology Analyser (URIT Medical Electronic Co Ltd, Guangxi, China).

### Data analysis

Data analyses were performed using Stata® 12.1(StataCorp LP, TX77845, USA) and EpiInfo™ 7 (CDC, Atlanta, USA). The data set was checked for logical inconsistencies, illegal codes, omissions and improbabilities by tabulating, summarising, describing and plotting variables. Missing observations were excluded because they constituted a small random proportion.

Our main outcomes of interest included: liver enzyme elevation (LEE) or transaminitis defined as ALAT > 45U/L and/or ASAT > 42U/L at the end of the study on day 15 of follow-up on ART; low haemoglobin level (anaemia) defined as haemoglobin level < 10g/dL at day 15 after ART initiation. This haemoglobin cut-off point of 10g/dL was based on the median value for the cohort irrespective of gender and age. Leucopenia was considered as an absolute white blood cell count < 4.0 × 10^3^ cells/μL while thrombocytopenia meant an absolute platelet count < 1.5 × 10^5^ cells/μL. The explanatory variables examined in our analyses included: age, gender, body weight, marital and educational statuses, alcohol intake, smoking, use of ITN, WHO clinical stage of HIV disease, CD4 count, baseline haemoglobin and transaminases, pregnancy status, ARV regimen and other medications. No factor was considered as an *a priori* confounder or effect modifier.

Summary statistics were presented as proportions for categorical variables and as means (standard deviations) or medians (IQR: Inter-quartile range) for continuous variables. Paired t-test analyses were used to examine the differences in means before and after ART. The McNemar’s X^2^ test (if discordant pairs ≥20) or the Fisher’s Exact test (if discordant pairs < 20) were used to compare proportions from paired data. A multivariable logistic regression model with random effects to account for correlated data was used to pick up factors independently associated with transaminitis or anaemia. Adjusted odd ratios (aOR) and their 95% confidence intervals (95% CI) were obtained. The p-values for hypotheses testing were calculated from Wald or likelihood ratio tests (LRT). A p-value <0.05 was considered significant.

## Results

### Characteristics of participants at baseline

The study enrolled patients initiating ART and returning to the clinic two weeks later for their first routine laboratory monitoring. The results of 154 adults are reported of whom 105 (68.2%) were females (Table 1). Their median age was 40 years (IQR: 33–50.5) with a median CD4 count of 204.5 cells/μl (IQR: 105–283.5). Late clinical presentation (WHO stages III and IV) was observed in 90 (65.7%) of cases with the commonest conditions being the constitutional syndrome (48.9%), lung disease (24.5%) and skin problems (6.7%). Over half (52.6%) reported ever using alcohol, 60 (39%) said they were using ITNs while just 19 (12.3%) declared they were current smokers. Only eight (7.8%) had no formal education and about half of them were married. The median ALAT level was 15U/L (IQR: 9.5-23) while the median ASAT level was 17U/L (IQR: 10–25). Moderate elevation of liver enzymes (>3×ULN) was observed in only 3 cases and severe elevation (>5×ULN) in none. The red cell indices were characterised by a median haemoglobin concentration of 10.6g/dl (IQR: 9.3-11.7) and a median mean cell volume of 82fl (IQR: 75–86). The median leucocyte and thrombocyte counts were respectively 5.2 × 10^3^ cells/ μl (IQR: 3.7-7.1) and 2.5 × 10^5^ cells/μl (IQR: 2.0-3.0). With lamivudine (3TC) constantly present in all the first line triple-combination antiretroviral regimens, zidovudine (AZT) completed the nucleotide reverse transcriptase inhibitor (NRTI) backbone in 79 (53.4%) cases while nevirapine (NVP) in 88 (59.5%) patients was the preferred non-nucleotide reverse transcriptase inhibitor (NNRTI) option.

**Table 1:** **Baseline characteristics of study participants**

Characteristics	Number (%)	Characteristics	Number (%)
**Gender**		**Absolute leucocyte count (/μl)**	
Female	105 (68.2)		
Male	49 (31.8)	<4000	36 (27.1)
Total	154 (100)	≥4000	97 (72.9)
		Total	133 (100)
**Age group (years)**		**Absolute platelet count (/μl)**	
<30	19 (12.5)		
30-49	93 (61.2)	<150000	19 (13.9)
≥50	40 (26.3)	≥150000	118 (86.1)
Total	152 (100)	Total	137 (100)
**Marital status**		**Fasting blood sugar (mg/dl)**	
Single	47 (32.2)		
Married	73 (50.0)	<126	130 (94.2)
Divorced	4 (2.7)	≥126	8 (5.8)
Widowed	22 (15.1)	Total	138 (100)
Total	146 (100)		
**Education level**		**NRTI-based regimen**	
None	8 (7.8)	AZT-based	79 (53.4)
Primary	33 (32.4)	D4T-based	2 (1.3)
Secondary	57 (55.9)	ABC-based	1 (0.7)
Higher	4 (3.9)	TDF-based	66 (44.6)
Total	102 (100)	Total	148 (100)
**Alcohol use**		**NNRTI-based regimen**	
No	73 (47.4)		
Yes	81 (52.6)	NVP-based	88 (59.5)
Total	154 (100)	EFV-based	60 (40.5)
		Total	148 (100)
**Smoking**		**Other medications**	
No	135 (87.7)	Tuberculosis	5 (3.25)
Yes	19 (12.3)	Haematinics	76 (49)
Total	154 (100)	Cotrimoxazole	133 (86.4)
		Fluconazole	7 (4.5)
		Traditional medicines	1 (0.7)
**Pregnancy**		**CD4 count (cells/μl)**	
No	144 (93.5)	<350	134 (93.1)
Yes	10 (6.5)	≥350	10 (6.9)
Total	154 (100)	Total	144 (100)
**ITN use**		**ALAT (U/L)**	
No	94 (61.0)	<45	136 (94.4)
Yes	60 (39.0)	≥45	8 (5.6)
Total	154 (100)	Total	144 (100)
**WHO clinical stage**		**ASAT (U/L)**	
I	26 (19.0)	<42	132 (91.7)
II	21 (15.3)	≥42	12 (8.3)
III	83 (60.6)	Total	144 (100)
IV	7 (5.1)		
Total	137 (100)		
**Weight (kg)**		**Haemoglobin (g/dl)**	
<60	62 (43.7)	<10	47 (32.4)
≥60	80 (56.3)	≥10	98 (67.6)
Total	142 (100)	Total	145 (100)

ALAT = alanine aminotransferase, ASAT = aspartate aminotransferase, ITN = insecticide treated nets, NRTI = nucleotide reverse transcriptase inhibitor, NNRTI = non-nucleotide reverse transcriptase inhibitor, ABC = abacavir, D4T = stavudine, AZT = zidovudine, TDF = tenofovir, EFV = efavirenz, NVP = nevirapine, WHO = World Health Organisation.

### Comparison of laboratory measurements before and after the second week of ART

**Figure 1:**
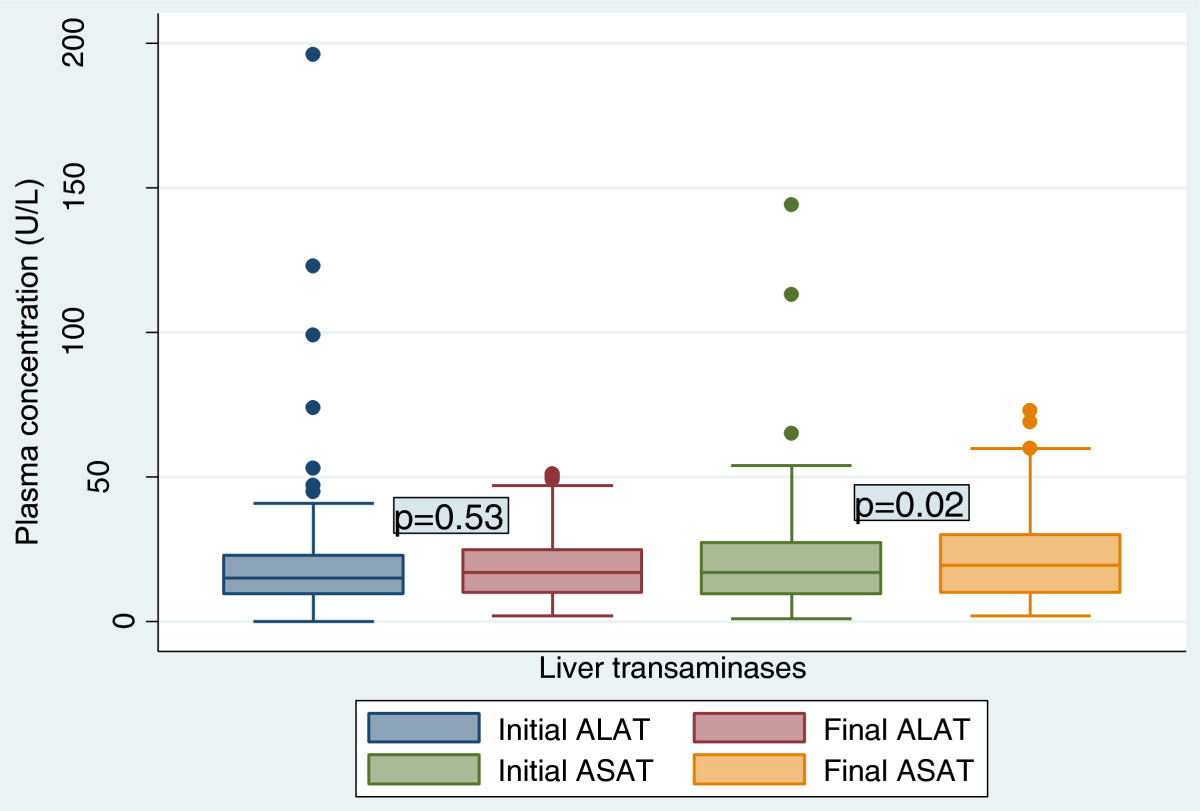
**Evolution of liver enzymes before and after two weeks of antiretroviral therapy initiation.**

**Figure 2:**
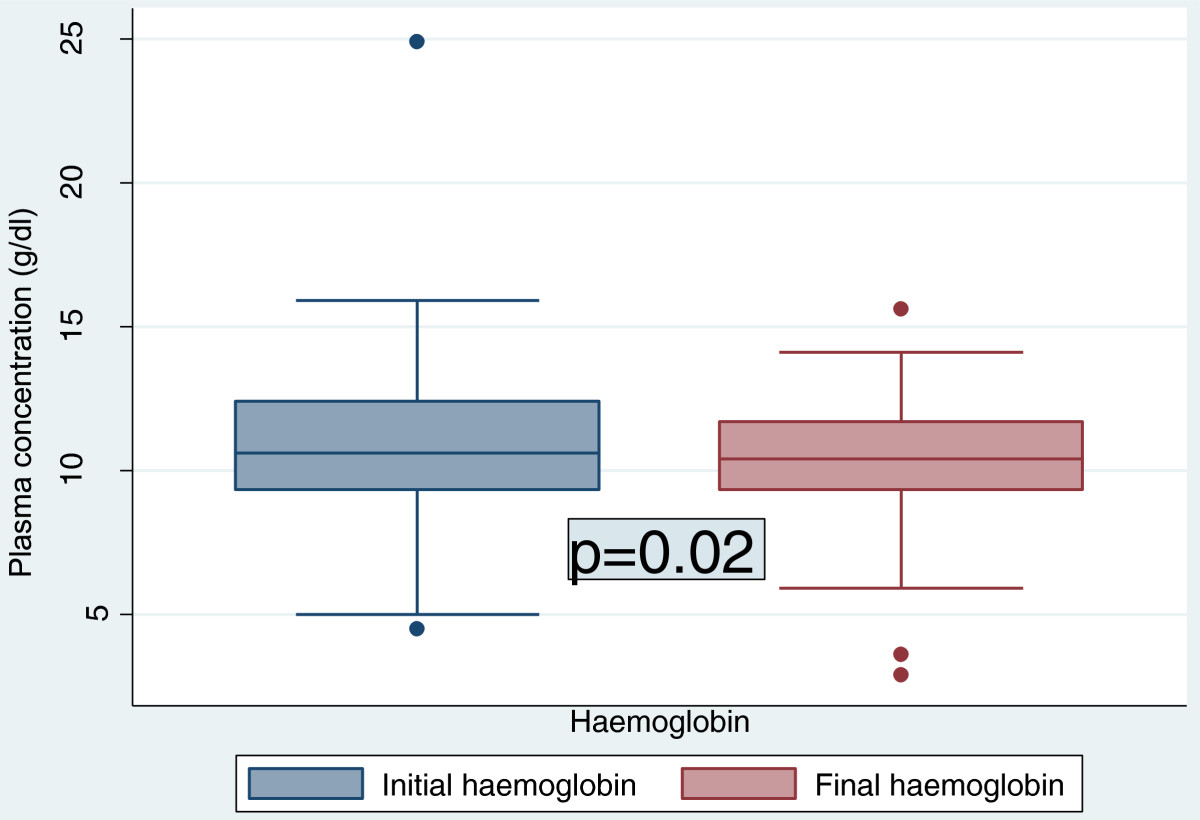
**Evolution of haemoglobin level before and after two weeks of antiretroviral therapy initiation.**

The duration on ART was 14 days equivalent to the study duration. Figure 1 shows that there was no evidence that there was a true rise in the mean ALAT levels from 17.87 ± 20.48 U/L at entry to 19.25 ± 12.01 U/L at follow-up (p=0.53). The evidence that the increase in the mean ASAT level from 17.32 ± 11.87 U/L to 21.02 ± 14.12 U/L was strong enough (p=0.02). ALAT and ASAT were correlated (r=0.31, p=0.0001). There was a small but significant fall in the mean haemoglobin concentration from 10.86 ± 2.63 g/dl to 10.36 ± 1.92 g/dl (p=0.02) [Figure 2] accompanied by a significant increase in the mean red cell volume from 79.62 ± 7.93 fl to 83.49 ± 9.13 fl (p=0.0004). While there was a very strong evidence of a drop in the total white blood cell count of 1.37 ± 3.62 × 10^3^ cells/ μl, the decrease in the total platelet count of 0.03 ± 1.18 × 10^5^ cells/ μl was not significant (Table 2).

**Table 2:** **Comparison of biochemical and haematological measurements before and after antiretroviral treatment**

Laboratory measurements	Pre-ART Mean (SD)	15^th^Day Post-ART Mean (SD)	Mean Difference (Pre-ART minus Post-ART)	95% CI of mean difference	p-value from paired t-test
**ALAT (U/L)**	17.87 (20.48)	19.25 (12.01)	−1.37 (23.10)	−5.73-2.99	0.53
**ASAT (U/L)**	17.32 (11.87)	21.02 (14.12)	−3.70 (16.61)	−6.84- -0.56	**0.02**
**Haemoglobin (g/dl)**	10.86 (2.63)	10.36 (1.92)	0.50 (2.29)	0.08-0.92	**0.02**
**Mean cell volume (fl)**	79.62 (7.93)	83.49 (9.13)	−3.86 (8.98)	−5.95- -1.78	**0.0004**
**Absolute leucocyte count (10** ^**3**^ **/μl)**	6.21 (3.44)	4.84 (1.76)	1.37 (3.62)	0.66-2.09	**0.0002**
**Absolute platelet count (10** ^**5**^ **/μl)**	2.72 (1.07)	2.69 (1.14)	0.03 (1.18)	−0.20- 0.25	0.82

ART = antiretroviral therapy, ALAT = alanine aminotransferase, ASAT = aspartate aminotransferase, CI = confidence interval.

### Prevalence and determinants of transaminitis and cytopenia after ART initiation

**Figure 3:**
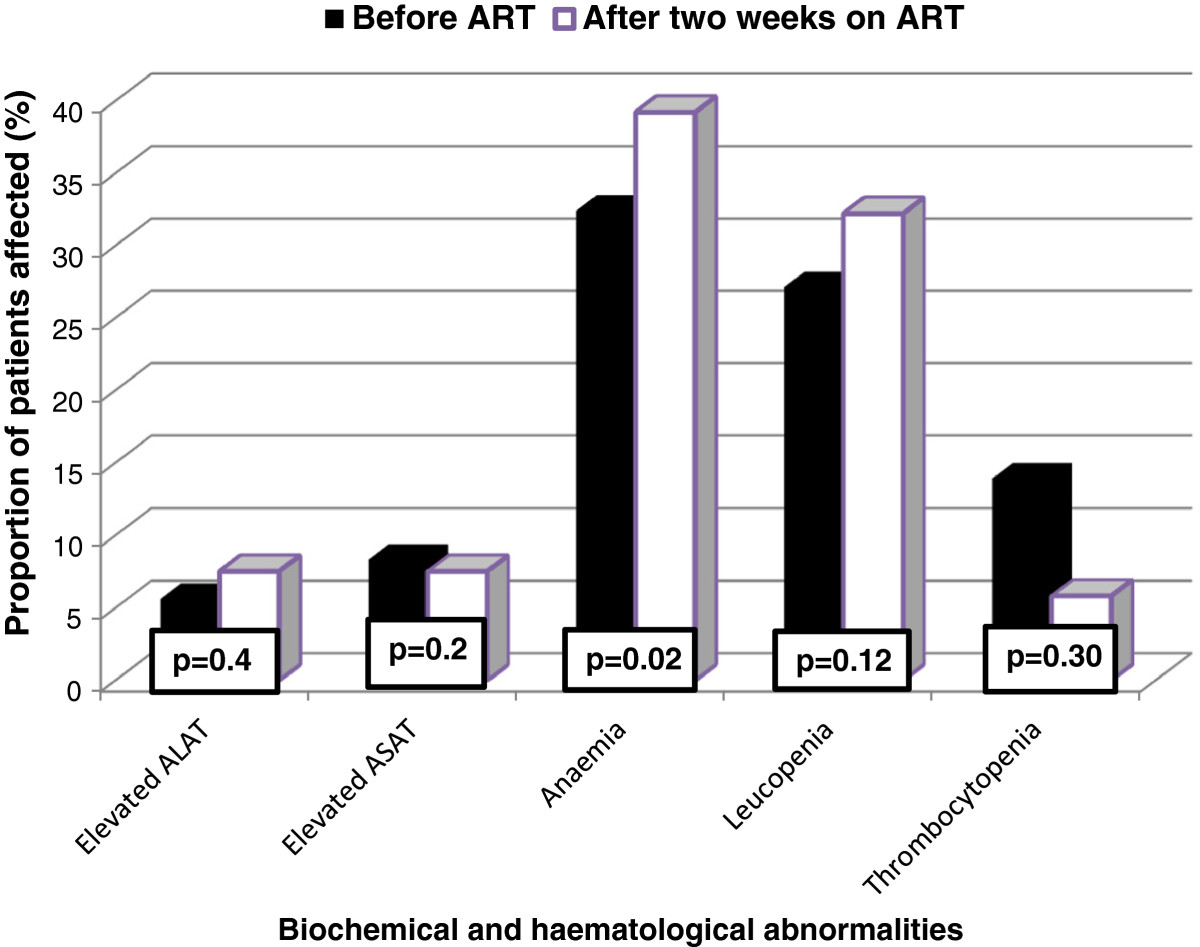
**Prevalence of liver enzyme elevation and cytopenia at baseline and at follow-up.**

Elevated transaminases or transaminitis was observed in 8.3% of the patients before starting ART and in 7.5% of them after two weeks of treatment with no significant difference between the proportions (Figure 3). Moderate elevation (>3×ULN) and severe elevation (>5×ULN) were recorded in none of the cases. The frequency of anaemia before treatment was 32.4% and rose significantly to 39.2% at the end week 2 of treatment (p=0.02). The prevalence of leucopenia at entry was 27.1%, increasing to 32.2% at follow-up. Though drastic, the fall in the occurrence of thrombocytopenia from baseline (13.9%) to follow-up (5.8%) was probably a chance finding (p=0.3).

Fasting blood sugar and ALAT were correlated (r=0.34, p < 0.0001).

There was some evidence that stavudine containing regimens were most likely to induce hepatotoxicity compared with other NRTI-based regimens (aOR=36.52, 95% CI: 1.44-924.38, p=0.029) as Table 3 depicts.

**Table 3:** **Predictors of early liver enzyme elevation and anaemia after two weeks of antiretroviral therapy**

Characteristics	Crude odd ratios (95% Confidence Interval)	p-value	Adjusted odd ratios (95% Confidence Interval)	p-value
**Early liver enzyme elevation**				
**NRTI-based regimens**				
AZT-based	1		1	
D4T-based	**18.3 (0.91-370.28)**	**0.058**	**36.52 (1.44-924.38)**	**0.029**
ABC-based	1		1	
TDF-based	1.83 (0.42-8.07)	0.423	3.43 (0.61-19.21)	0.160
**Fasting blood sugar (mg/dl)**				
<126	1		1	
≥126	6.40 (1.02-40.12)	**0.047**	6.26 (1.00-39.30)	**0.50**
**Early anaemia**				
**Baseline haemoglobin (g/dl)**				
≥10	1		1	
<10	25.6 (8.61-76.17)	**<0.0001**	60.08 (13.36-270.20)	**<0.0001**
**Body weight (kg)**				
<60	1		1	
≥60	0.32 (0.12-0.88)	**0.027**	0.28 (0.09-0.83)	**0.022**
**Use of ITN**				
No	1		1	
Yes	4.41 (1.43-13.64)	**0.010**	4.60 (1.35-15.71)	**0.015**
**NRTI-based regimens**				
AZT-based	1		1	
D4T-based	1		1	
ABC-based	1		1	0.252
TDF-based	3.38 (1.56-7.32)	**0.002**	2.01 (0.61-6.64)	
**Use of haematinics**				
No	1		1	
Yes	3.74 (1.75-8.00)	**0.001**	1.06 (0.32-3.49)	0.921
**Marital status**				
Single	1		1	
Married	0.43 (0.18-0.99)	**0.049**	0.54 (0.14-2.01)	0.357
Divorced	1		1	
Widowed	0.47 (0.15-1.53)	0.212	0.81 (0.15-4.42)	0.804

ITN = insecticide treated nets, NRTI = nucleotide reverse transcriptase inhibitor, NNRTI = non-nucleotide reverse transcriptase inhibitor, ABC = abacavir, D4T = stavudine, AZT = zidovudine, TDF = tenofovir, EFV = efavirenz, NVP = nevirapine.

In univariable analysis, baseline anaemia, tenofovir containing regimens, marital status, low body weight, use of haematinics and ITNs were associated with anaemia. After correcting for other covariates in a multivariable model, patients with anaemia at presentation were very likely to remain anaemic after two weeks of therapy (aOR=60.08, 95% CI: 13.36-270.20, p < 0.0001). A body weight of 60kg and above was associated with a 72% reduction in the risk of anaemia at day 15 of ART (aOR=0.28, 95% CI: 0.09-0.83, p=0.02). Subjects who reported using an ITN were associated with a 4.6-fold increase in the odds of developing anaemia (aOR=4.60, 95% CI: 1.35-15.71, p=0.015).

## Discussion

This study has demonstrated neither a statistically nor a clinically significant liver enzyme elevation (LEE) after two weeks of ART initiation. Though there was a statistically significant rise in the frequency of anaemia after a fortnight of receiving ART, the presence of anaemia prior to ART initiation was the key determinant.

The prevalence of liver enzyme elevation after two weeks of ART was 7.5% in our cohort all of which were mild and asymptomatic elevations (<3×ULN). The findings are consistent with the reported prevalence rates of 1% to 10% in developed countries [10–13] and of <1% to 7% across cohorts from developing countries [14–17]. The variability in the frequency of ART-induced liver injury has been attributed to differences in the choice of ARV regimen used, comorbid conditions, concomitant or alternative therapies, host genetics, available laboratory monitoring technology. The small sample size, the laboratory reference intervals or cut-off points and the short duration of exposure to ART in our cohort may further explain the variability in the occurrence of LEE.

Given that the duration on ART was constant and that the difference between baseline and follow-up LEE was insignificant, the incidence of LEE that can be attributable to ART is also likely to be negligible. The effect of HIV infection on liver enzymes might wholly be responsible for the observed LEE. Similarly, the before and after ART mean difference in liver enzymes may have statistical importance in this study but clinically, the mean transaminase levels are just about a half of their upper limits of normal. This therefore means that a two-week course of ART has little or no clinical impact on liver enzymes. Moreover, the stavudine-containing regimen that has been isolated in our study as the single most important contributor of LEE has completely been phased out in Cameroon. Consequently, monitoring liver enzymes for drug toxicity at the end of the second week of ART may not be necessary. The implication of this finding would be economical: on one hand, patients who pay out of their pockets for these tests would now have more money to be diverted to other treatment related expenses like transportation and feeding; on the other hand, the country program would equally stand to benefit as some of the already constraint human and material resources will be spared. An economic evaluation will obviously be required to justify the cost-effectiveness of such a recommendation in perspective.

The prevalence of anaemia after two weeks of ART was 39.2%. This rate is higher than the rates of 3% to 12% in studies from Nigeria, Cote d’Ivoire, Haiti and India though the latter were related to long-term zidovudine-based therapies [14, 15, 18–20]. The rate is however lower than either the 76% observed at the 6^th^ week of using a winniecure antiretroviral drug in a Nigerian cohort [21] or the 46% reported in a Ghanaian population receiving ART for at least 3 months [22]. We observed a 0.5g/dl drop in mean haemoglobin from baseline to the end of second week of follow-up. A similar decrease of 0.5g/dl in the first three months of using AZT-based regimens has been observed in large cohorts across countries in sub-Saharan Africa, Asia-Pacific, and Central and South America [23] but Mgogwe et al. had reported a decrease of up to 1.3g/dl during six months of ART in Tanzania [24]. Conversely, a rise in mean haemoglobin levels has been experienced in patients receiving non-AZT regimens [23] and after a long term use of successful treatment combinations with or without AZT [21, 25, 26].

The 0.5g/dl drop in the mean haemoglobin level was statistically significant but may have little clinical importance after two weeks of treatment when the mean haemoglobin had remained satisfactorily above 10g/dl. Unlike liver enzymes, the significant rise of about 6.8% in the prevalence of anaemia after a fixed two-week period from baseline might indicate a true increase in the incidence of anaemia that can be attributed to ART assuming that other predictors of anaemia remained unchanged during this very short exposure time. The high prevalence of anaemia at baseline and during follow-up coupled with a significant fall in the total leucocyte count in this cohort, and the fact that anaemia is known to have been associated with a 2.5-fold increase in the risk of mortality in the same but a larger cohort [7], means that early monitoring of haematological indices for ARV drug-induced myelosuppression is justifiable.

Baseline anaemia was frequent and was a major predictor of anaemia at follow-up in this study and several others [2, 8, 23, 26]. Unlike other cohorts, our study duration of 15 days was too short to allow for a complete red blood cell turnover of 120 days, therefore the persistence of anaemia was inevitable. Late presentation was prevalent (65.7%) in our study population and is likely to be responsible for the high prevalence of anaemia. This overwhelming contribution of baseline anaemia at initiation of ART suggests that a targeted monitoring of ART for haematological toxicity could be an alternative for the on-going routine monitoring.

Low body weight (LBW) which is indicative of low body mass index (BMI) was associated with more than a 3.5-fold increase in the odds of anaemia after initiating ART. LBW is a surrogate marker of advanced HIV infection or poor nutritional status characterised by depleted iron and protein stores required for the production of haemoglobin.

Use of ITN is well known to reduce the impact of malaria and anaemia in developing countries [27]. Paradoxically, in this study users of ITNs had a higher risk of anaemia than non-users. We could neither ascertain the effectiveness, nor ownership nor the duration of the supposed use of ITN. Information bias is very likely in this case. An unknown factor distal or proximal to ITN such as socio-economic status or geographical location of users may be responsible for this strong association between ITN use and risk of anaemia.

Though tenofovir-containing regimens and the use of haematinics (iron ± folate ± vitamin B12) were very strong determinants of anaemia in univariate analysis, they were correctly ruled out after adjusting for other factors and for correlation in data. This is due to the fact that patients presenting with anaemia at baseline were given haematinics and in order to avoid worsening of anaemia they were more likely to receive non-AZT regimens especially TDF because D4T was being phased-out. This selection bias was responsible for the apparent association. It is has now been demonstrated that baseline severe anaemia should not preclude use of zidovudine in antiretroviral-eligible patients in resource-limited settings [26].

Our study had some draw backs. The sample size was small and made up of patients returning voluntarily to the clinic and able to afford for the laboratory investigations at the 15^th^ day of starting ART. All patients receiving ART at the clinic are usually informed of the necessity for the first visit which also has a clinical component. This suggests that persons with low socio-economic status or living far off from the clinic might have been excluded. There is no reason however to believe that the subgroup that did not have a laboratory monitoring at day-15 of ART (but had at least a clinical monitoring) must have experienced a different toxicity profile because drug toxicity is essentially a biological phenomenon. The study’s retrospective design made it liable to incompleteness of information collected. For example, we could not obtain data about co-infection with the hepatitis viruses B and C which are prevalent in this setting.

## Conclusion

In conclusion, at the end of the second week of ART initiation, there has been no significant rise in mean liver enzymes and thus scheduling their routine monitoring at that point in time could be skipped. Fortunately, the ART program has also scheduled their monitoring where appropriate at the 30^th^, 60^th^, 90^th^ day and so on [5]. Conversely, the drop in mean haemoglobin level during the second week of ART has had little clinical importance but the high prevalence of anaemia warrants a targeted monitoring as an alternative to routine laboratory monitoring particularly for the high risk population with baseline anaemia and low body weight.

## Authors’ information

CEB (MD, MSc, DLSHTM) is Chief Medical Officer of the Bare Sub-divisional Medical Centre and a visiting physician at the HIV Clinic of the Nkongsamba Regional Hospital in Cameroon.

CS is a medical laboratory scientist at the Regional Hospital of Nkongsamba in Cameroon.

HD is the data clerk of the HIV clinic at The Regional Hospital of Nkongsamba in Cameroon.

PSB (MD) is a surgeon and the Director of the Nkongsamba Regional Hospital.

BK (MD, Sc.D.) is The Head of Department of Public Health at The University of Douala, Cameroon, Board Chairman of The Nkongsamba Regional Hospital.

## Authors’ original submitted files for images

Below are the links to the authors’ original submitted files for images. Authors’ original file for figure 1 Authors’ original file for figure 2 Authors’ original file for figure 3
