# Supplementary figures and images for: Evaluating the utility of early laboratory monitoring of antiretroviral-induced haematological and hepatic toxicity in HIV-infected persons in Cameroon
Source: BMC Infect Dis. 2014 Sep 25;14:519. doi: 10.1186/1471-2334-14-519 (PMC4262146; doi:10.1186/1471-2334-14-519)

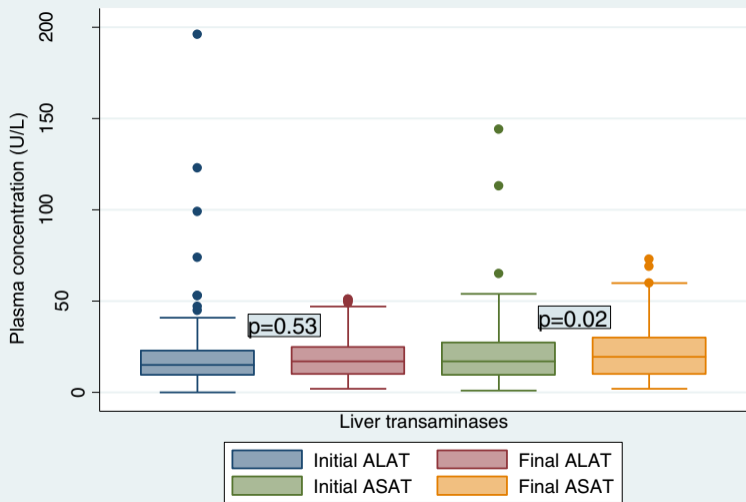

Supplement: Supplementary file 1 — Authors’ original file for figure 1 [file 12879_2014_3840_MOESM1_ESM.pdf]

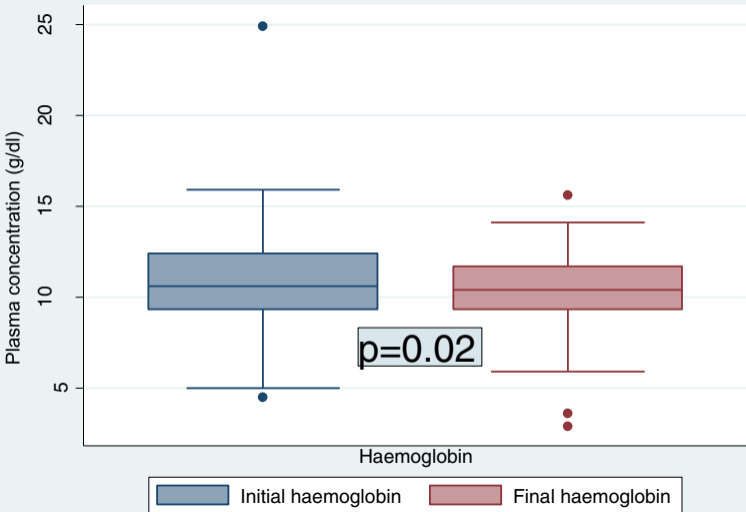

Supplement: Supplementary file 2 — Authors’ original file for figure 2 [file 12879_2014_3840_MOESM2_ESM.pdf]

■ Before ART    □ After two weeks on ART

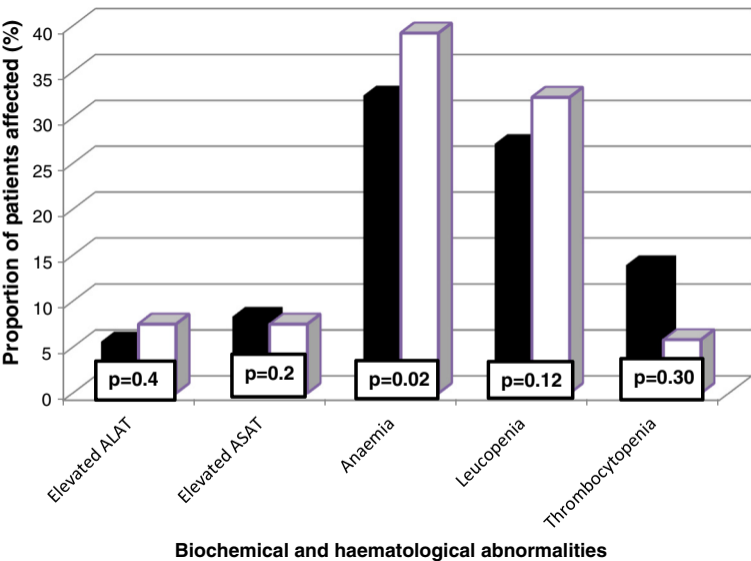

Supplement: Supplementary file 3 — Authors’ original file for figure 3 [file 12879_2014_3840_MOESM3_ESM.pdf]
